# Suggesting a new design for multileaf collimator leaves based on Monte Carlo simulation of two commercial systems

**DOI:** 10.1120/jacmp.v11i3.3101

**Published:** 2010-06-15

**Authors:** Sanaz Hariri, Majid Shahriari

**Affiliations:** ^1^ Department of Radiation Medicine Engineering Shahid Beheshti University Tehran Iran; ^2^ Department of Radiation Application Shahid Beheshti University Tehran Iran

**Keywords:** Monte Carlo, MCNP4C, multileaf collimator (MLC), intensity‐modulated radiation therapy (IMRT)

## Abstract

Due to intensive use of multileaf collimators (MLCs) in clinics, finding an optimum design for the leaves becomes essential. There are several studies which deal with comparison of MLC systems, but there is no article with a focus on offering an optimum design using accurate methods like Monte Carlo. In this study, we describe some characteristics of MLC systems including the leaf tip transmission, beam hardening, leakage radiation and penumbra width for Varian and Elekta 80‐leaf MLCs using MCNP4C code. The complex geometry of leaves in these two common MLC systems was simulated. It was assumed that all of the MLC systems were mounted on a Varian accelerator and with a similar thickness as Varian's and the same distance from the source. Considering the obtained results from Varian and Elekta leaf designs, an optimum design was suggested combining the advantages of three common MLC systems and the simulation results of this proposed one were compared with the Varian and the Elekta. The leakage from suggested design is 29.7% and 31.5% of the Varian and Elekta MLCs. In addition, other calculated parameters of the proposed MLC leaf design were better than those two commercial ones. Although it shows a wider penumbra in comparison with Varian and Elekta MLCs, taking into account the curved motion path of the leaves, providing a double focusing design will solve the problem. The suggested leaf design is a combination of advantages from three common vendors (Varian, Elekta and Siemens) which can show better results than each one. Using the results of this theoretical study may bring about superior practical outcomes.

PACS number: 87.56.nk, 87.55.K, 87.56.N

## I. INTRODUCTION

The aim of radiation therapy is to eradicate tumor cells without seriously damaging normal tissue, especially without seriously damaging radiation‐sensitive structures. In addition, tailoring the treatment volume as closely as possible to the target volume or, in other words, distribute dose conformal to the target volume or conformal radiation therapy is a main objective. There are cases where conventional conformal treatment planning and delivery techniques fail and using intensity‐modulated radiation therapy (IMRT) can solve these problems. The three most frequently used techniques of delivering IMRT are the step‐and‐shoot approach, the dynamic approach and physical compensators. To generate intensity‐modulated photon fields, a modern linear electron accelerator (linac) equipped with a multileaf collimator (MLC) can be used.

Analytical expressions can be used to find the optimal set of performance parameters for a MLC system.^(^ ^1^ ^)^ Alternatively, a numerical trial and error approach can be used, such as ray tracing^(^ ^2^ ^,^ ^3^ ^)^ or a Monte Carlo based approach.^(^ ^4^ ^–^ ^7^ ^)^ Monte Carlo techniques are widely used in all medical physics applications. Monte Carlo simulation of radiation transport is considered a highly accurate method of radiation therapy dose calculation. It makes no assumptions regarding radiation equilibrium, so it can be accurate for every small field sizes and in regions of disequilibrium.^(^ ^8^ ^)^ Also, the Monte Carlo code can actually trace the photons or electrons through the MLC, even in a moving one.

Several papers have addressed the creation of phase space distributions (PSDs) for modeling the patient‐independent photon beam radiotherapy accelerator output.^(^ ^9^ ^,^ ^10^ ^)^ Each of these works used EGS4‐based Monte Carlo transport. Calculations of the PSD and the patient dependent portion of the beam line have been performed using the BEAM user code,^(^ ^11^ ^)^ too. Different versions of MCNP are additional useful tools for radiotherapy dosimetry. MCNP4A was used by DeMarco et al.^(^ ^12^ ^)^ for radiotherapy dose calculations, while Lewis et al.^(^ ^13^ ^)^ and Kim et al.^(^ ^14^ ^)^ used MCNP4B and Aaronson et al.^(^ ^15^ ^)^ used MCNP4C for this purpose.

The dosimetric characteristics of MLC systems have also been evaluated. Kim et al.^(^ ^14^ ^)^ have compared the leaf end transmission and leakage radiation for Varian 80 and 120 leaf MLCs using Monte Carlo simulations. Huq et al.^(^ ^16^ ^)^ have compared three MLC systems including Varian, Elekta and Siemens by means of dosimetric films. They have studied some dosimetric parameters including penumbra width, radiation leakage and isodose curves, and they have concluded that there is no perfect MLC system that can be recommended. Topolnjak et al.^(^ ^1^ ^)^ have presented an analytical approach for optimizing the leaf design of a MLC based on the penumbra width and compared their design with Elekta, Varian and Siemens collimator designs.

The work reported here compares several dosimetric parameters of two most common MLC systems, Elekta and Varian. Some optimizations were done based on five dosimetric parameters on these systems and an optimum design was suggested to achieve the best characteristics in the mentioned parameters. Finally the three MLC systems were compared. The dosimetric parameters including leakage as a function of field size, beam hardening by the MLC, leaf tip transmission, inter‐ and intra‐leaf transmission and penumbra width are calculated using the Monte Carlo method.

As the intent of this paper is to focus on leaf design, MLC models were assumed to have the same leaf width of 10 mm at isocenter and 80‐leaf pairs. Since the 80‐ and 120‐leaf Varian MLCs do not show a significant difference in design and dosimetric parameters with each other,^(^ ^14^ ^,^ ^17^ ^)^ we used the 80‐leaf MLC to keep the uniformity of designs. Also the same thickness and distance from the source was supposed for all of them and equal to what is seen in a Varian system.

## II. MATERIALS AND METHODS

### A. Monte Carlo

#### A.1 The MCNP4C code

MCNP4C is a general‐purpose, three‐dimensional Monte Carlo code which can be used for neutron, photon and electron or coupled neutron/photon/electron transport.^(^ ^18^ ^)^ For photon transport, the code takes into account incoherent, coherent scattering and pair production. Also, the possibility of fluorescent emission after photoelectric absorption and bremsstrahlung are included. The continuous slowing down approximation energy loss model is used for electron transport. To follow an electron through a significant energy loss, the MCNP4C code breaks the electron's path into many steps. For electron transport, MCNP4C addresses the sampling of bremsstrahlung photons at each electron substep. MCNP4C was used for the transport of radiation through the accelerator, MLC and phantom. The phantom was divided into several voxels in order to determine the dose distribution in it. The energy deposited in voxels was scored by means of tally *F8 that scores the deposited energy of photons in the desired volumes per source particle. To obtain the absorbed dose, the energy deposited in each voxel was divided by its mass.

Monte Carlo‐based dose calculation algorithms typically separate the dose computation problem into two stages.^(^ ^19^ ^)^ The first stage is computation through the patient‐independent part of the beam‐line apparatus, up to a plane just upstream of the accelerator jaws. This is referred to as the “patient‐independent beam‐line Monte Carlo simulation” or the “patient‐independent simulation”. This computation is performed only once for a given treatment machine energy, with the resulting particle coordinates (energy, location, direction and particle type) stored or modeled in the form of a phase space distribution (PSD) for use in the second stage, the patient specific portion of the simulation. In this stage, transport takes place through modeled patient‐specific beam‐line devices (jaw, blocks and/or wedges) and the patient, with dose being scored in the phantom as a patient. This stage is referred to as the “treatment planning Monte Carlo simulation”. For the MCNP4C run, 450 million electrons are incident upon the target, resulting in about 25 million photons at the PSD plane location at 17 cm from the target. The simulation results show an uncertainty of 5% which is acceptable for MCNP code.

#### A.2 Beam‐line model

**Figure 1:**
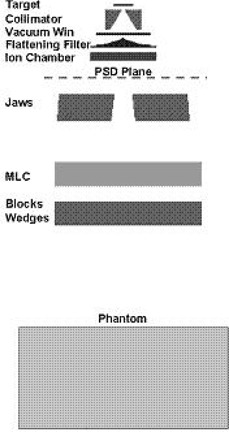
Schematic layout of a Varian 2100c linear accelerator treatment head modeled for phase space generation.

A basic schematic layout of the accelerator is shown in Fig. 1. Items included in the patient‐independent beam‐line simulation are the bremsstrahlung target (with target backing), the conical primary collimator, the vacuum window and the flattening filter. In the patient‐independent beam‐line simulation (upstream of the beam defining jaws), the beam‐line components are cylindrically symmetric.

The 6 MV X‐ray mode of the Varian 2100c accelerator is modeled. The electron beam incident upon the target is modeled as a parallel circular beam with a 0.1 cm radius.^(^ ^9^ ^,^ ^20^ ^)^ The incident electrons possessed an energy distribution with a Gaussian outline with a center at 6.5 MeV for 6 MV beam and FWHM of 0.5 MeV (Varian Oncology Systems Ltd.). As with the study by Siebers et al.,^(^ ^19^ ^)^ the photon energy transport cutoff was set to 0.01 MeV, while the electron kinetic energy cutoff was 0.189 MeV. The simulation runs in the patient‐independent stage used no variance reduction while, for the patient‐specific portion, variance reduction techniques like geometry splitting and Russian roulette were used.

#### A.3 MLC leaf model

##### A.3.1 Varian system

**Figure 2:**
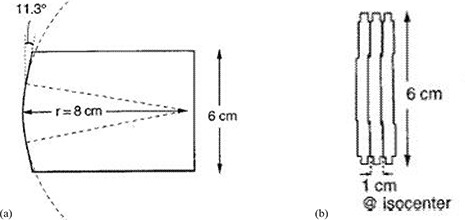
Views of the Varian leaves from the side (a) and from the front (b). The leaf face is rounded near the center with a radius of curvature of 8 cm. The outer portions of the face are straight, 11.3° off the vertical.

For each Varian MLC, two banks of independent tungsten alloy leaves face each other and travel linearly perpendicular to the beam central axis. Orthogonal to the direction of motion, the leaf edge is parallel to the beam ray line from the target. Figure 2 shows a cross‐sectional and front view of the 80‐leaf Varian MLC. All details of the leaf design were included in the Monte Carlo geometry, including the tongue‐and‐groove used to reduce radiation leakage through interfaces between adjacent leaves and the complex rounded leaf tip.

The central 3 cm portion of each leaf end is circular with a radius of curvature of 8.0 cm. Beyond this, the leaf end is straight and at an angle of 11.3° relative to the vertical axis.^(^ ^21^ ^)^ The MLC leaf material is a sintered tungsten alloy. These tungsten alloys have densities in the range of 17.0 to $18.5\;g/{\text{cm}}^{3}$; $17.7\;g/{\text{cm}}^{3}$ was chosen as a base.^(^ ^14^ ^)^

##### A.3.2 Elekta system

**Figure 3:**
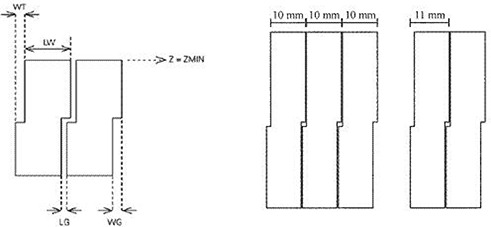
Design of the Elekta multileaf collimator, described by the parameters: tongue width (WT), groove width (WG), leaf gap (LG), and leaf width (LW) along with their values (on the right).

The number and material of the Elekta leaves were taken similar to Varian system. The Elekta multileaf collimator has curved leaf ends and a stepped design for the leaf sides (Fig. 3). The curvature of leaf ends were considered to be the same as Varian's. The projection of the leaf pitch in the isocentric plane is 1.0 cm^(^ ^22^ ^)^ and similar to Varian's. The Elekta multileaf collimator is placed 29.8 cm below the target and has a thickness of 7.5 cm. However, in this study we consider it similar to Varian MLC in order to make a fair comparison of leaf design. More detailed information of the Elekta multileaf collimator can be found in the papers by Jordan et al.^(^ ^23^ ^)^ and Sykes et al.^(^ ^24^ ^)^ Provided that the total transmission dose is a function of one‐hundred‐thousandth of the air gap size^(^ ^5^ ^)^ and for consistency with two other MLC systems, the air gap between the Elekta MLC leaves was not taken into account.

##### A.3.3 Suggested system

**Figure 4:**
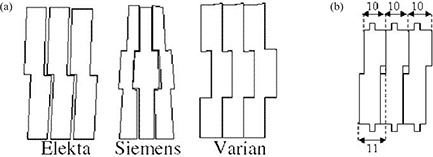
Schematic diagram of the leaf end of various leaves from different manufacturers showing the differences in leaf design (a)^(^ ^16^ ^)^; schematic diagram of the suggested leaf design (b).

Because there is little difference between Siemens and Varian leaf designs, the simulation of Siemens system was ignored. Also, the Siemens MLC produces reduced tongue‐and‐groove effect compared to the other two collimators (Elekta and Varian).^(^ ^16^ ^)^ The main differences between leaf designs of these systems are the middle leaf (Fig. 4) as well as the double‐focused design of Siemens MLC^(^ ^25^ ^)^ in comparison with single‐focused leaves of Elekta and Varian. The advantage of a double‐focusing leaf design is that all effects are nearly independent of field position.^(^ ^26^ ^)^ The transmission through the leaf tip has a contribution to the total penumbra for the Elekta and Varian MLCs, but not for the Siemens MLC. However, the total penumbra for the Siemens MLC is bigger than for Varian.^(^ ^16^ ^)^ This is due to the fact that the leaves are closer to the source (geometric penumbra).^(^ ^1^ ^)^ As a result, if the same distance for all leaves from the source is assumed, double‐focusing design is advantageous over single‐focusing, except from mechanical design complication point of view.

Considering the results obtained from simulation of Varian and Elekta MLC leaf designs and the preference of double‐focused design of MLC leaves to single‐focused and non‐focused, a suggested design was proposed. The suggested leaf design has a stepped side like Elekta and tongues on top and bottom of the leaf to guide it through the rails like Varian MLC. In contrast with Elekta and Varian systems but similar to the Siemens, the leaf end is flat and, using a circular motion path, produces double‐focusing design with less leakage compared with Elekta and Varian (as will be discussed later). All other properties were assumed similar to Varian MLC system. Figure 4(b) shows the proposed leaf design.

### B. Studies performed

#### B.1 Leakage as a function of field size

To determine the MLC radiation leakage as a function of field size, a MLC blocked field was configured such that the leaf tips were situated on the central axis, at an SAD of 100 cm. The MLC leaf in this case blocked the area defined by the jaws. Simulations made with the MLC blocked fields were normalized to open fields (without MLC) of the same field size. Four standard field sizes were simulated. Positioning the leaf tips on the central axis in contrast with other studies performed by Huq et al.,^(^ ^16^ ^)^ Kim et al.^(^ ^14^ ^)^ and LoSasso et al.^(^ ^21^ ^)^ was intentional, to make a comparison between leakages from rounded and flat leaf tips. Although it results in more leakage than what was calculated and measured by Kim et al., the profiles are in a good agreement with each other from an appearance point of view. The energy deposited in the phantom was recorded using a standard energy deposition tally in a volume produced by $3\times 3\;{\text{cm}}^{2}$ square with 2 cm depth in the central axis direction.

#### B.2 Beam hardening by the MLC

The effect of the MLC on the beam hardness was determined by scoring the photon energy spectra in the confined area by the MLC at 95 cm SSD for a jaw‐defined $10\times 10\;{\text{cm}}^{2}$ open field and the same field blocked by the MLC. To illustrate the effect of this beam hardening, percent depth dose profiles were also scored in $5\times 5\times 0.1\;{\text{cm}}^{3}$ voxels for a water phantom located at 100 cm SSD using MCNP4C.

#### B.3 Leaf tip transmission

The characteristics of the radiation field edge formed by the leaf tips was studied by positioning the leaf tips on the central axis and using MCNP4C to compute dose in a water phantom with $0.1\times 4\times 4\;{\text{cm}}^{3}$ voxels at an SSD of 95 cm. The simulation was performed with a $10\times 10\;{\text{cm}}^{2}$ field and was normalized to open field.

#### B.4 MLC transmission

In addition to leakage as a function of field size, the transmission for a constant field size of $10\times 10\;{\text{cm}}^{2}$ as a function of depth was determined. This simulation was performed by energy deposition calculation in a water phantom situated at 100 cm SSD and in 2 cm depth steps. The results were normalized to open filed defined by jaws.

Taking into account tongue‐and‐groove effect in different designs, in addition to transmission as a function of depth, midleaf and interleaf transmissions were calculated. Simulations were made for a $10\times 10\;{\text{cm}}^{2}$ field defined by the upper and lower jaws, with the MLC in the open and closed positions. The ratio of energy deposition values in these two conditions in a water phantom with 4×0.$2\times 4\;{\text{cm}}^{3}$ voxels is the transmission. For the measurements on the axis for “closed” MLC, in order to avoid leakage between the rounded ends, the junction between the opposed leaves was placed off axis and under the jaw with an offset of 10 cm.

#### B.5 Penumbra width

The penumbra measurement in the direction of leaf motion was performed with *F8 tally in voxels with $0.1\times 5\times 2\;{\text{cm}}^{3}$ volume placed perpendicular to the beam central axis in a water phantom. The phantom was placed at 100 cm source‐to‐axis distance (SAD) at the depth including maximum dose, $d_{\max}$, for a $10\times 10\;{\text{cm}}^{2}$ field size. Simulation was made for the situation where a $10\times 10\;{\text{cm}}^{2}$ field size is positioned symmetrically with respect to the central axis. The profile was normalized to the value at the centre of the $10\times 10\;{\text{cm}}^{2}$ field. The penumbra width is the region between 80% and 20% dose. Similar to other simulations, the MLC leaves were modeled perpendicular to the central axis for the three systems.

## III. RESULTS & DISCUSSION

After all studies were performed for Varian and Elekta MLC systems, they were done for the proposed leaf design, too. Finally, the comparison between these three systems is shown as separate profiles for each experiment.

### A. Leakage as a function of field size

**Figure 5:**
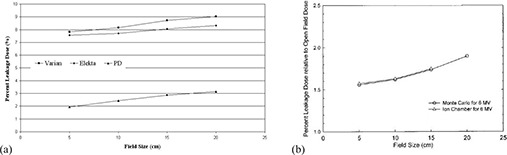
Radiation leakage dose in a $3\times 3\times 2\;{\text{cm}}^{3}$ volume centered on the beam central axis for the three leaf designs (a); radiation leakage dose at 5 cm depth in a water phantom at 95 cm SSD from MLC blocked fields for 6 MV as a function of the field size (b).^(^ ^14^ ^)^

Figure 5(a) shows radiation leakage in a water phantom as a function of field size for Varian, Elekta and the suggested 80‐leaf MLCs. Recall that the MLC fully blocks the field during this test and the field size is determined by the setting of the jaws. Figure 5(b) ^(^ ^14^ ^)^ can be used as a benchmark for the calculated dose profile which shows less leakage than ours because of positioning the leaves under the jaws and excluding the leaf tip transmission. Leakage from Varian is more than from Elekta and it is more than from the proposed design (PD). For $10\times 10\;{\text{cm}}^{2}$ field, the leakage through PD MLC is 29.7% and 31.5% order of the Varian and Elekta's, respectively.

### B. Beam hardening by the MLC

**Figure 6:**
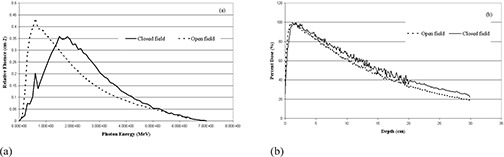
Relative photon fluence in central $30\times 40\;{\text{cm}}^{2}$ area at 95 cm SSD for a $10\times 10\;{\text{cm}}^{2}$ field fully blocked and unblocked by the Elekta MLC (a); percent depth doses (%DD) in the water phantom for $10\times 10\;{\text{cm}}^{2}$ field with and without Elekta MLC blocking for 6 MV photon beam (b).

The preferential removal of lower energy photons by the MLC is apparent for the 6 MV beam in all three designs and their effects are approximately similar. The effect of the MLC on the beam quality is demonstrated in Fig. 6(a) which shows the photon fluence on the central $30\times 40\;{\text{cm}}^{2}$ surface of a $10\times 10\;{\text{cm}}^{2}$ field for Elekta MLC. The fluence plots are normalized so the total area under the curve equals one.

**Figure 7:**
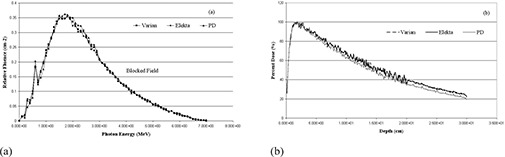
Comparison of relative photon fluence on central $30\times 40\;{\text{cm}}^{2}$ area at 95 cm SSD for a $10\times 10\;{\text{cm}}^{2}$ field fully blocked by Varian, Elekta and the proposed design (PD) MLCs (a); comparison of percent depth doses (%DD) in the water phantom for $10\times 10\;{\text{cm}}^{2}$ field size with MLC blocking for the three designs (b).

Also, the effects of the photon beam hardening on the depth dose characteristics of Elekta MLC are shown in Fig. 6(b). By using more than double number of photon histories to achieve less statistical uncertainty, the 6 MV MLC blocked beam shows more penetration, with the percent depth dose (%DD) at 10 cm increasing about 7%, from 61.2%±0.8% for the open beam to 68.3%±2.0% for the MLC blocked field. On the other hand, this effect is less pronounced for Varian and the proposed system which show an increase of 5% and 2%, respectively. It can cause more penetration for Elekta MLC systems than the other two and, as a result, higher doses will be delivered to the tissues beyond the target volume. The comparison of fluence outlines in the blocked field for three designs is shown in Fig. 7(a). There is no significant difference between the three systems. Figure 7(b) demonstrates the comparison between percent depth doses (%DD) in the three MLCs where $10\times 10\;{\text{cm}}^{2}$ field is fully blocked. It can be understood that the Varian and Elekta systems have similar percent depth dose to each other; however, the proposed design shows a bit less value than the other two.

### C. Leaf tip transmission

**Figure 8:**
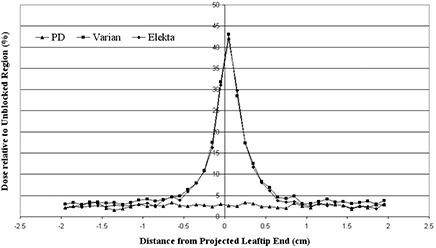
Leaf tip dose profiles at a depth of 5 cm in a phantom at 95 cm SSD for the 80‐leaf MLC leaf tips on the central axis for $10\times 10\;{\text{cm}}^{2}$ field size.

Leaf tip transmission profile at a depth of 5 cm in a water phantom at 95 cm SSD for the 80‐leaf MLC leaf tips centered on the field is shown in Fig. 8 for the three designs. The x‐axis is given as the distance from the central axis. Each profile was normalized to the corresponding $10\times 10\;{\text{cm}}^{2}$ open field. Leaf tip transmission for Varian MLC is almost similar to Elekta's, except in the wings of the plot which shows more transmission. The MLC with the suggested design has a flat profile because it has flat leaf tips that result in a very little leaf tip transmission (about 2.5%).

### D. MLC transmission

**Figure 9:**
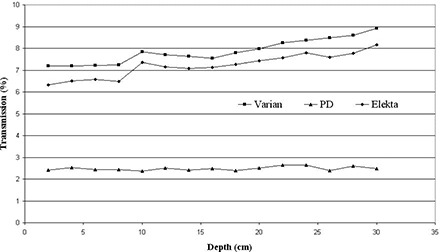
The average of midleaf and interleaf transmissions simulated at isocenter in the water phantom for 6 MV photons for $10\times 10\;{\text{cm}}^{2}$ field size.

The average of midleaf transmission and interleaf leakage is shown in Fig. 9 for 6 MV photon beams, normalized to the output of the open field as a function of depth. The transmission is calculated for a $10\times 10\;{\text{cm}}^{2}$ centered field over depths from the surface to 30 cm, which shows an average of 7.86%, 7.20% and 2.48% for Varian, Elekta and the suggested MLCs, respectively. The transmission increases with depth of measurement, while the proposed design does not show the same profile. This may be due to the flat leaf tips that results in statistical fluctuations about 0.27% around the mean value.

**Figure 10:**
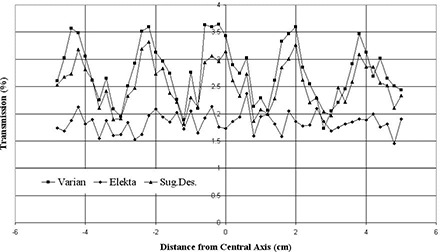
The midleaf and interleaf transmissions calculated at isocenter in water phantom for a $10\times 10\;{\text{cm}}^{2}$ field size defined by the jaws and an offset of 10 cm for the leaf tips.

The midleaf and interleaf transmission profile obtained for a $10\times 10\;{\text{cm}}^{2}$ field is displayed in Fig. 10. The peaks are due to the interleaf leakage, while valleys show the midleaf transmission. The average transmissions are 2.78%±0.54%, 1.83%±0.18% and 2.53%±0.40% for Varian, Elekta and the suggested design, respectively, while accounting for one standard deviation confidence interval. The proposed design shows a profile between Varian and Elekta MLC leaf designs. The range of transmission over the central portion of the MLC was from 1.81% at midleaf to 3.32% between leaves for suggested MLC.

### E. Penumbra width

**Figure 11:**
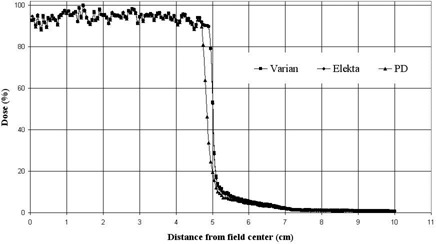
Comparison of the penumbra width for the three collimators when the leaves are positioned at the edge of a centered $10\times 10\;{\text{cm}}^{2}$ field for 6 MV beams.

Figure 11 shows a comparison of the penumbra width for 6 MV beams for the three devices. The leaves are positioned at the edge of a centered $10\times 10\;{\text{cm}}^{2}$ field and with the voxels of 2 cm depth, which includes the depth of maximum buildup. The Varian and Elekta collimators show the sharpest dose gradient in the region between 80% and 20% dose (penumbra) and the proposed design has the largest penumbra width. There is not a significant difference among the Varian and Elekta MLC systems and the penumbra widths are 1.51 and 1.48 mm, respectively – consistent with the results from the Huq et al.^(^ ^16^ ^)^ study which has shown that the penumbra widths are within 2.0 mm of each other.

Although this value is 2.52 mm for the proposed design, taking into account the curved motion path of the leaves, which is the main component of a double‐focusing design, will solve the problem.

## IV. CONCLUSIONS

The properties of radiation leakage from Varian and Elekta multileaf collimators have been studied using Monte Carlo simulations. Using these results, a leaf design was proposed. The results of these studies are useful for developing an optimum MLC leaf design.

Monte Carlo accurately predicts the increase in MLC leakage with field size. As it is seen from the previous section, the proposed design led to a very smaller leakage compared with Varian and Elekta leaf designs. The MLC substantially modifies the photon energy spectrum at 6 MV. The spectrum hardening results in hardening of the beam depth dose. However, this effect in the proposed leaf design is negligible and less important from a clinical point of view to make a deeper penetration.

In contrast with Varian and Elekta systems, there is no leaf tip transmission for suggested design as a result of flat leaf ends. On the other hand, transmission as a function of depth is smaller, too. Moving the leaf tips to avoid the leaf tip transmission shows a better comparison between PD and the commercial systems. Although it shows a value between those two, using a heavier material such as depleted Uranium can result in a similar profile with Elekta's.

The only disadvantage about the proposed design is the penumbra width. As a double‐focusing design needs a circular motion path, which was not considered in our simulations, this deficiency was shown.

In summary, the proposed leaf design for MLC systems shows promising results in the field of IMRT and guarantees the as‐low‐as‐reasonably‐achievable (ALARA) rule.
